# The test-retest reliability of the H-Reflex and swaymeter in women aged 30–65 years

**DOI:** 10.1371/journal.pone.0314437

**Published:** 2025-06-23

**Authors:** Rosiered Brownson-Smith, Jack Carr, Kai T. Fox, Samuel T. Orange, Nicola Cresti, John Saxton, John Temesi

**Affiliations:** 1 Department of Nutritional Sciences, Faculty of Life Sciences & Medicine, King’s College London,; 2 Department of Sport, Exercise and Rehabilitation, Faculty of Health and Life Sciences, Northumbria University, Newcastle-upon-Tyne, United Kingdom; 3 Research Centre for Physical Activity, Sport and Exercise Sciences, Coventry University, Coventry, United Kingdom; 4 School of Biomedical, Nutritional and Sport Sciences, Faculty of Medical Sciences, Newcastle University, Newcastle-upon-Tyne, United Kingdom; 5 Newcastle University Centre for Cancer, Newcastle University, Newcastle-upon-Tyne, United Kingdom; 6 Northern Centre for Cancer Care, The Newcastle upon Tyne Hospitals NHS Foundation Trust, Newcastle-upon-Tyne, United Kingdom; 7 School of Sport, Exercise & Rehabilitation Sciences, University of Hull, United Kingdom; Universita Politecnica delle Marche Facolta di Ingegneria, ITALY

## Abstract

**Background:**

The H-reflex can objectively assess sensorimotor integration relevant to proprioception and postural control. Chemotherapy-induced peripheral neuropathy (CIPN) is a common and potentially dose-limiting side effect among breast cancer patients undergoing taxane-based treatments, leading to proprioception loss and balance issues, and an increased risk of falling. This study evaluated the between-day test-retest reliability of neurophysiological measures and postural sway, measured via swaymeter in a demographically similar population to those affected by breast cancer.

**Methods:**

25 women aged 30–65 years were assessed on two occasions, approximately two weeks apart. The study measured between-day test-retest reliability of the soleus H-reflex and M-wave and postural sway, measured by the swaymeter.

**Results:**

The soleus maximum H-reflex amplitude (H_max_) and maximum M-wave amplitude (M_max_) exhibited good to excellent (ICC = 0.92, 95% CI: 0.82–0.96) and moderate to excellent (ICC = 0.81, 95% CI: 0.62–0.91) between-day reliability, respectively. The H_max_/M_max_ showed poor to good between-day reliability (ICC = 0.63, 95% CI: 0.31–0.82), and H-reflex latency demonstrated moderate to excellent between-day reliability (ICC = 0.83, 95% CI: 0.65–0.92). The between-day test-retest reliability of anteroposterior (AP) displacement and total sway across the two testing sessions was poor to moderate (ICC = 0.37, 95% CI: −0.03–0.66 and ICC = 0.42, 95% CI: 0.04–0.70, respectively).

**Conclusions:**

H-reflex and M-wave measures are reliable for between-day assessments. However, sway measures via swaymeter were less reliable, indicating a need for more robust tools to evaluate balance.

## Introduction

Peripheral neuropathies, often resulting from chronic conditions or neurotoxic treatments like chemotherapy, can impair balance and neuromuscular function [1]. Chemotherapy-induced peripheral neuropathy (CIPN) is a neuropathic disorder common in those receiving taxane-based chemotherapy for breast cancer [2–4]. Loss of proprioception resulting from CIPN often manifests as balance and/or gait disorders, with balance being linked to a higher risk of falling and an impact on daily life activities [1,5]. Ia fibre damage caused by neurotoxic chemotherapy may interfere with proprioceptive cues, reducing the reliability of afferent feedback via sensory axons [1].

The H-reflex is an electrically induced reflex analogous to the spinal stretch reflex. The soleus is an important muscle for maintaining postural control and requires appropriate processing of afferent feedback in response to sensory input from muscle spindles [6]. The H-reflex can be used to assess the excitability of the Ia afferent spinal loop at rest and during functional tasks such as postural control [6]. Eliciting the soleus H-reflex via electrical stimulation of the tibial nerve allows measurement of synaptic transmission efficacy, integrating sensory presynaptic inputs to produce a post-synaptic motor response. Electrical stimulation of the tibial nerve also causes direct stimulation of the efferent motor fibres and the ensuing muscle response, measured via electromyography as the M-wave. The maximal H-reflex activation (H_max_) normalised to the maximal M-wave (M_max_) allows for estimation of the contribution of the motoneuron pool to the H-reflex [7]. Changes in the H-reflex and M-wave over time may identify underlying changes related to the development of CIPN. Specifically, a smaller soleus H_max_/M_max_ has previously been reported in women with CIPN post-chemotherapy for breast cancer versus a healthy control group [1] while other research has identified differences more generally in plantar flexor H-reflex parameters between individuals with peripheral neuropathy and healthy controls [8].

Previous studies have found the test-retest reliability of the H-reflex in the soleus while standing to be reliable, with intraclass correlation coefficients (ICC) indicating high consistency across repeated measures, ranging from moderate to high reliability (ICC values from 0.7 to 0.9) across different postures [9–11]. These studies included a combination of male and female participants with ages ranging from 20–50 years. Women in the age group at higher risk for developing breast cancer may experience age-related changes, such as reduced muscle mass and muscle weakness [12]. However, it must be noted that these changes are less pronounced in the soleus older of women who are able to walk without assistance [13]. In comparison, younger women are typically characterised by lower muscle strength, neurological function, and proprioception compared to younger men [14,15]. Given breast cancer’s prevalence among women over 50 years in the UK, and its status as a leading cause of death for those aged 35–49 years [16,17], and the observed sex differences in soleus H-reflex [18], it is critical to understand measurement variability in a demographically relevant population. Additionally, diurnal fluctuations in H-reflex amplitude in rat [19] and primate [20] models, and humans [21] necessitate assessment of the within-day reliability of the measurement.

To evaluate the relationship between potential changes in Ia afferent transmission at the spinal level and balance deficits, it is critical to have a reliable measure of postural stability in older women. The cost and space requirements of available accurate measures of sway, such as force plates and motion capture systems, mean that they are not always viable within a clinical setting. The swaymeter has been proposed as a low-cost, practical, reliable device to record body displacement at the waist level [22]. An increase in sway area can determine the deficit of balance control and be used to assess the risk of falls [23]. The swaymeter has previously been shown to be reliable in older (71–83 years) and younger adults (22–47 years) [22]. Given the potential relevance of this technique to assess sway in individuals with breast cancer, it is important to assess the test-retest reliability of this method in women of a comparable age.

The primary objective of this study was to evaluate the between-day test-retest reliability of the soleus H-reflex and M-wave in women aged 30–65 years. Secondary outcomes were to evaluate the between-day test-retest reliability of sway using a swaymeter, and within-day test-retest reliability of the H-reflex and M-wave and sway in women aged 30–65 years.

## Methods

### Sample participants

Twenty-six women (age: 43 years ± 10 years, height 165.9 ± 5.2 cm, body mass: 74.58 ± 13.71 kg) participated in this study after providing written informed consent. Women were recruited via convenience sampling from the North East of England between 4 May 2023 and 5 February 2024. The inclusion criteria were women aged 30–65 years who were able to understand written and verbal instructions in English. Exclusion criteria included cardiovascular, neurological, or respiratory illness, diabetes, musculoskeletal injury affecting the lower limbs, pregnancy, previous exposure to neurotoxic chemotherapy or radiotherapy, the precence of an internal electrical regulator, or the inability to elicit H_max_ or M_max_ responses. The study was conducted in accordance with the Declaration of Helsinki. However, its protocol was not registered in a database. University ethical approval was granted by the Northumbria University Ethics Committee.

### Sample size calculation

The sample size required was calculated using the hypothesis-testing approach [24], based on the primary study outcome. The threshold ICC value of 0.75 was set to indicate good reliability, based on established classification guidelines [25]. The true ICC was anticipated to be 0.9, derived from a previously reported ICC for the primary outcome (H-reflex) in the standing position [9]. Using these parameters, and assuming a significance level of 0.05 and power of 80%, the estimated sample size was N = 26 [26].

### Procedures

#### Study design.

**Fig 1:**
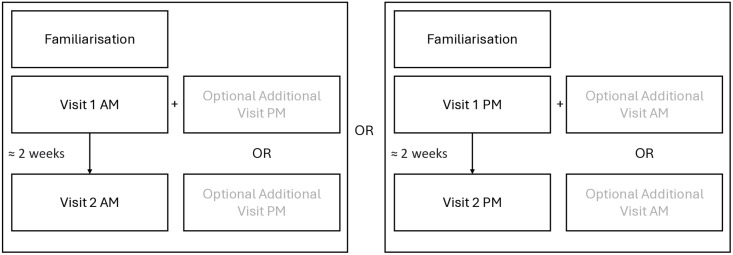
Study Schematic.

The study design was observational. Participants visited the lab on three or four occasions: once for familiarisation, at least 24 hours before the first test session, and then for two or three retest trials (Fig 1). During the familiarisation, participants were familiarised with electrical nerve stimulation of the tibial nerve so that they were comfortable standing while receiving the stimulations. Electromyography (EMG) was recorded to confirm the H-reflex and M-wave signals in the soleus.

The primary data collection took place during the initial test session (visit 1) and the retest session (visit 2) approximately two weeks later (15 ± 2 days). Visits 1 and 2 took place at the same time of day (either morning (6am - 11am) or afternoon (2 pm - 7 pm). Each data collection session included H-reflex/M-wave and recording of sway via the swaymeter. An additional third data collection session was used to assess diurnal fluctuations and was therefore scheduled for the alternative timeslot (either morning or afternoon) on the same day as either visit 1 or 2 (Fig 1). This session was optional to reduce participant burden.

### Eliciting the H-reflex/M-wave

Sensorimotor integration was assessed using H-reflexes and M-waves elicited by electrical stimulation (1-ms pulse duration) of the tibial nerve of the right leg via constant-current stimulator (DS7A, Digitimer, Welwyn Garden City, Hertfordshire, UK) and recorded by EMG in the soleus muscle [1,7] while standing in a bipedal stance, approximately shoulder width apart. To elicit the H-reflex and M-wave, a stimulus was delivered to the tibial nerve at the knee using a felt pad stimulating bipolar bar electrode (Neurospec AG, Stans, Switzerland). The tibial nerve was stimulated at a 10-s interstimulus interval. Beginning at low intensity (5 mA), the stimulus was increased in increments of 1 mA until 40 mA or a plateau in the M-wave amplitude was reached, whichever occurred later. The recording electrodes were placed on the soleus muscle, with the centre of the proximal electrode 5 cm below the distal border of the gastrocnemius [27] and the reference electrode placed on the right malleolus. Data were analog-to-digitally converted at a sampling rate of 2000 Hz using a PowerLab 8/35 data acquisition system and Quad Bio Amp (ADInstruments, Oxford, Oxfordshire, UK), with bandpass filter (5–500 Hz). Data were analysed offline using Labchart 8 (ADInstruments).

### Sway

The swaymeter recorded displacement of the body in the horizontal plane [22]. The swaymeter is an inflexible 40 cm rod with a vertically mounted pen at one end and belt for attachment at the other end. The rod was located at the level of the posterior superior iliac spine, with the belt sitting firmly on the lower back. The pen was at the same height and recorded the displacement of the body by tracing a line on a sheet of millimetre graph paper. Participants were asked to stand barefoot in bipedal stance, legs shoulder width apart, as still as possible without talking for 30 s.

### Data analysis

H-reflex and M-wave recruitment curves were recorded. The maximum H-reflex amplitude (H_max_) was expressed relative to the maximum M-wave amplitude (M_max_), defined as H_max_/M_max_. H-reflex latency was determined visually as the time between the stimulus and the first slope of the H-reflex at H_max_ [6].

Standing balance was quantified using three sway metrics: anteroposterior (AP) displacement, mediolateral (ML) displacement, and total sway path length. The sway path was traced on graph paper using a pen attached to the participant’s waist or harness, capturing postural movement during quiet standing. The total sway path length was calculated manually by counting the number of 1 mm² grid squares the pen traversed and summing their linear distances, approximating the total distance travelled by the centre of pressure. Peak-to-peak sway displacements in the AP and ML directions were determined by measuring the maximum excursion between the furthest points reached in each respective direction along the sway path. These values were extracted using a ruler aligned with the AP and ML axes to quantify the extreme ranges of postural movement [22].

### Statistical analysis

Descriptive data were presented as means and standard deviation. Normality of data was examined through visual inspection (histograms) and hypothesis testing (Shapiro-Wilks). The following parameters were not normally distributed: H_max_, H_max_/M_max_, H-reflex latency, AP displacement, ML displacement. Due to the positive skew, the data underwent log10 transformation [28]. Following the transformation, the data were rechecked for normality and were found to be normally distributed. A two-way random-effects, absolute agreement, single measurement intraclass correlation coefficient [ICC (2,1)] was used to determine the relative reliability of the measure between the two visits [29]. The significance of the ICC was evaluated using an F-test, and the associated p-value was reported. The size of ICC point estimates was interpreted as: poor (< 0.50), moderate (0.50 to 0.74), good (0.75 to 0.89), and excellent reliability (≥ 0.9), respectively [25]. In accordance with guidelines, interpretation of ICC included 95% confidence intervals (CI) [25]. Absolute reliability was examined by calculating standard error of measurement (SEM) and calculated as SEM = SD × (1– ICC), where SD is mean the standard deviation of both visits. SEM was then presented as a percentage of the overall mean (mean of the two visits) [30]. Minimal Detectable Change (MDC) was calculated using the formula MDC = 1.96 × √2 × SEM [31]. All P values below 0.05 were considered significant. All statistical analyses were conducted using SPSS for Windows (SPSS Inc., Chicago, IL, ver. 29.0).

## Results

### Between-day test-retest reliability

Table 1 shows the means and standard deviations of H_max_, M_max_, H_max_/M_max_, and H-reflex latency. One participant’s data was removed as M_max_ was not achieved during the testing session. The test-retest reliability of soleus H_max_ in the standing position was good to excellent (Table 2), moderate to excellent for M_max_, and poor to good for H_max_/M_max_. The H-reflex latency had moderate to excellent test-retest reliability (Table 2).

**Table 1:** Descriptive statistics for visit 1 and 2.

	Visit 1 (n = 25)	Visit 2 (n = 25)
**H**_**max**_ **(mV)**	2.88 ± 2.08	2.64 ± 1.94
**M**_**max**_ **(mV)**	9.84 ± 4.23	9.72 ± 3.89
**H**_**max**_ **/M**_**max**_	0.33 ± 0.27	0.28 ± 0.18
**H-**reflex **Latency (ms)**	29.0 ± 6.5	29.0 ± 6.5
**AP Displacement (mm)**	17 ± 7	19 ± 7
**ML Displacement (mm)**	14 ± 7	15 ± 9
**Total Sway (mm)**	73 ± 26	77 ± 29

Values are mean ± SD; n=25. Abbreviations: AP= anteroposterior, ML= mediolateral.

**Table 2:** Between-day and AM/PM test-retest reliability of H-reflex, M-wave, and Sway measures.

Between-day				
	**ICC [95% CI]**	**SEM (%)**	**MDC**	**F-test p-value**
**H**_**max**_ **(mV)**	0.92 [0.82, 0.96]	18.74	1.48	0.00
**M**_**max**_ **(mV)**	0.81 [0.62, 0.91]	17.52	4.77	0.00
**H**_**max**_ **/M**_**max**_	0.63 [0.31, 0.82]	44.96	0.38	0.00
**H-reflex Latency (ms)**	0.83 [0.65, 0.92]	9.16	7.36	0.00
**AP Displacement (mm)**	0.37 [−0.03, 0.66]	30.24	14.99	0.03
**ML Displacement (mm)**	0.14 [−0.26, 0.50]	49.56	19.53	0.25
**Total Sway (mm)**	0.42 [0.04, 0.70]	27.61	57.16	0.02
**AM/PM**				
	**ICC [95% CI]**	**SEM (%)**	**MDC**	**F-test p-value**
**H**_**max**_ **(mV)**	0.88 [0.38, 0.98]	30.44	3.31	0.00
**M**_**max**_ **(mV)**	0.76 [0.02, 0.96]	15.50	4.33	0.02
**H**_**max**_ **/M**_**max**_	0.73 [−0.05, 0.96]	29.17	0.28	0.03
**H-reflex Latency (ms)**	0.92 [0.52, 0.99]	2.66	0.00	0.00
**AP Displacement (mm)**	0.06 [−0.60, 0.66]	26.22	12.48	0.44
**ML Displacement (mm)**	0.36 [−0.35, 0.81]	47.36	16.26	0.15
**Total Sway (mm)**	0.37 [−0.34, 0.81]	26.41	53.76	0.14

ICC (alongside CIs), SEM, and F-test p-value are presented. Abbreviations: ICC = intraclass correlation coefficient, SEM = standard error of measurement, MDC = Minimal Detectable Change, CI = confidence interval, AP = anterioposterior, ML = mediolateral.

The means and standard deviations of AP displacement, ML displacement and total sway are shown in Table 1. The data for one participant was removed as they were unable to complete the test. The test-retest reliability of AP displacement and total sway was poor to moderate (Table 2). The ICC for ML displacement was not statistically significant.

### Diurnal fluctuations

The means and standard deviations of H_max_, M_max_, H_max_/M_max_ and H-reflex latency are shown in Table 3. Six participants performed the same-day AM/PM comparison for the neurophysiological assessments and were included in the analysis, less than for sway analysis due to two participants unable to complete the session and a hardware issue affecting one data file. AM/PM test-retest reliability of soleus H_max_, M_max_ and H_max_/M_max_ was poor to excellent. H-reflex latency had moderate to excellent test-retest reliability (Table 2).

**Table 3:** Descriptive statistics for AM/PM visits.

	AM (n = 6)	PM (n = 6)
**H**_**max**_ **(mV)**	3.97 ± 4.19	3.87 ± 2.96
**M**_**max**_ **(mV)**	11.06 ± 3.89	9.08 ± 2.28
**H**_**max**_ **/M**_**max**_	0.31 ± 0.19	0.39 ± 0.21
**H-reflex Latency (ms)**	30.0 ± 2.5	30.0 ± 3.0
	**AM (n = 9)**	**PM (n = 9)**
**AP Displacement (mm)**	19 ± 4	16 ± 5
**ML Displacement (mm)**	14 ± 9	11 ± 5
**Total Sway (mm)**	75 ± 24	72 ± 27

Values are mean ± SD. Abbreviations: AP= anterioosterior, ML= mediolateral.

The means and standard deviation of the AP displacement, ML displacement and total sway are shown in Table 3. The ICCs for all sway measures in this comparison of AM-PM measures were not statistically significant (Table 2).

## Discussion

This study investigated the reliability of the soleus H-reflex and M-wave and sway in women aged 30–65 years. The primary outcome was the between-day test-retest reliability of these measures. The soleus H_max_/M_max_ demonstrated poor to good between-day test-retest reliability. H_max_ and M_max_ exhibited good to excellent and moderate to excellent reliability, respectively. H-reflex latency displayed a moderate to excellent level of between-day test-retest reliability. The sway measures were less reliable. Both total sway and AP displacement between the two days demonstrated a poor to good level of reliability. H_max_ had poor to excellent and H-reflex latency moderate to excellent within-day reliability, whereas M_max_ and H_max_/M_max_ had poor to excellent within-day reliability.

### H-reflex and M-wave

Research that has considered the reliability of the H-reflex in functional positions such as standing, has shown that the intersession reliability of the H_max_ is good [32]. Although this finding indicates a lower level of reliability than presented within the current study, they measured intersession reliability over a series of 5 days using a one-leg balancing task, rather than bipedal standing. In contrast, Handcock et al (2001) assessed H_max_ during quiet bipedal standing and reported data that are consistent with our results, showing excellent reliability across several trials, similarly separated over two sessions. Diurnal changes in H_max_ have previously been observed in animals [19,20] and humans [21]. However, the current study did not observe a difference between the morning and afternoon and found the reliability of the soleus H_max_ in the standing position to be moderate to excellent. This suggests that there are limited changes in H-reflex amplitude across the day in this population.

The reliability of the soleus H_max_/M_max_ in bipedal standing between two days had not been studied in a sample of women of this age group. This measure demonstrated good to excellent reliability in neutral (ICC = 0.86), plantarflexion (ICC = 0.96), and dorsiflexion (ICC = 0.84) positions, with excellent reliability during plantar flexion in young adult males and females [33]. The intersession reliability of the H_max_/M_max_ during treadmill walking has also been evaluated revealing a good intraclass correlation (ICC = 0.89), in a young adult population [34], consistent with the findings of the current study (ICC = 0.77). While the between-day ICC value for H_max_/M_max_ was categorised as moderate, the 95% CI ranged from poor to good, with lower between-day reliability than either H_max_ or M_max_. Despite the lower between-day reliability for H_max_/M_max_, accounting for changes at the muscle by normalising H_max_ to M_max_ is essential when performing longitudinal studies such as during cancer treatment, particularly where changes in afferent Ia fibre excitability may be expected.

In line with the current findings, H-reflex latency has previously been shown to be robust when recorded in a standing position across multiple days [9]. In the current study the H-reflex latency measure also demonstrated moderate to excellent test-retest reliability across a single day when measurements were taken in the morning and afternoon. These results align with current published data showing within-day reliability coefficients in the range of 0.96 to 0.99 for H-reflex latency [9]. However, it is important to note that the sample size for the morning-afternoon analysis in the present study was small, as the additional visit was optional for participants. Further research is required to investigate the possible implications of the time of day of assessment, with this as the primary outcome.

### Sway

The results from this study indicate that the swaymeter may not produce consistent results when assessed between days. Our study found that total sway and AP displacement between two testing sessions demonstrated a poor to moderate level of reliability. There is currently no published research on the reliability of the swaymeter for measuring body sway in this age group of women. However, the reliability of the swaymeter has previously been compared with sway and displacement data from a force plate, the current ‘gold standard’ for measuring postural control [26]. In that study, the AP displacement, ML displacement and total path length as measured by a swaymeter were shown to have a moderate correlation with the force plate data in older and younger adults [22]. Test-retest reliability of the swaymeter has also been assessed in children, showing similar levels of consistency across time, with moderate reliability for both AP and ML displacement when assessed during sessions separated by a week. This study demonstrated that AP displacement was strongly correlated with a motion capture system (r = 0.979) [35].

Considering the above research, it is challenging to attribute the poor reliability observed for sway measures in this study to limitations inherent in the swaymeter device. Therefore, it could be argued that the measurement issues may also stem from the characteristics of the population studied and the testing protocol used. Unlike force platforms, the swaymeter relies on a mechanical arm and pen system to trace movement, which can be more susceptible to noise and user-dependent variability. Minor inconsistencies in device setup, alignment, or attachment to the participant’s lower back could introduce measurement error between sessions. This is especially noteworthy as placement was dependent on palpation. Furthermore, using a single trial may have reduced reliability compared to protocols that average multiple trials to account for natural fluctuations in postural sway.

The limited available research and the large confidence intervals observed in the current study indicate that further investigation is needed to confirm the reliability and usefulness of results derived from the swaymeter in adults. Future studies should aim to increase the sample size to achieve more precise reliability estimates. Sway measures have the potential to detect balance impairments resulting from neurological disorders, and therefore, could be useful for clinical diagnostics [26]. However, the lack of reliability demonstrated by the swaymeter indicates that there remains a need for a reliable and accessible measure of sway in clinical populations. Although studies have demonstrated that sway as measured by the ‘gold standard’ force platform is a reliable tool for assessing balance in healthy individuals across repeated assessments [36], this method incurs a high initial investment, significant space requirements and complex data analysis that pose logistical challenges for clinical practice.

### Limitations

Firstly, there are limitations within the experimental method. The consistency of placement for the stimulating and recording electrodes was maintained by recording a number of measurements with palpated reference points. The research team decided not to mark the electrode placement, in order to reduce the burden of maintaining the placement over two weeks. While palpated reference points can provide consistency, they may not offer the same level of accuracy as marked placements and can increase inter-session variability [37]. Secondly, the additional visit to assess diurnal test-retest reliability was optional to aid recruitment and prioritise the primary outcome of between-day test-retest reliability. This resulted in a small sample size being available to assess the latter. Therefore, additional research is required with diurnal variability as the primary outcome.

There were also sample limitations in the primary reliability analysis. The calculated target sample size was 33, but only 26 participants were recruited due to resource constraints. The study also did not account for potential participant dropouts in the overall sample size estimation, which may have impacted statistical power if it weren’t for the low attrition rate observed. Although several significant differences were observed, comparisons related to diurnal reliability did not reach statistical significance. This was likely due to limited statistical power, stemming from the optional nature of the additional assessment visit. Additionally, although the population was chosen to reflect those most at risk for a breast cancer diagnosis, and therefore CIPN, it does not represent those most at risk for falls, with older adults aged 75 years and older being up to five times more likely to fall than people aged 65 years or less [38]. Furthermore, our sample did not include individuals with active breast cancer undergoing chemotherapy. As a healthy population was used, it remains possible that unknown disease-related factors in clinical groups could further impact reliability. Therefore, additional validation of reliability may be warranted for this group.

Finally, the authors acknowledge the benefits of averaging multiple trials to investigate reliability within postural control studies. However, a single trial was used to reflect real-world applicability better and to reduce participant burden.

## Conclusion

While H_max_, M_max_, H_max_/M_max_ and H-reflex latency can be reliably used, the future use of the swaymeter as a cost-effective, accessible method to monitor balance control may be limited in women aged 30–65 years. While this study provides valuable insights into the test-retest reliability of the H-reflex in this population, longitudinal studies are needed to investigate the feasibility and acceptability of performing these measures regularly in those undergoing a chemotherapy regimen.
